# Teplizumab in Type 1 Diabetes Mellitus: An Updated Review

**DOI:** 10.17925/EE.2023.19.2.7

**Published:** 2023-10-06

**Authors:** Simran Thakkar, Aditi Chopra, Lakshmi Nagendra, Sanjay Kalra, Saptarshi Bhattacharya

**Affiliations:** 1. Department of Endocrinology, Indraprastha Apollo Hospitals, New Delhi, India; 2. Department of Endocrinology, Manipal Hospital, Bengaluru, India; 3. Department of Endocrinology, JSS Medical College, Mysuru, India; 4. Department of Endocrinology, Bharti Hospital, Karnal, Haryana, India

**Keywords:** Anti-CD3 monoclonal antibody, immunomodulation, regulatory T cells, teplizumab, tolerance, type 1 diabetes mellitus

## Abstract

Type 1 diabetes mellitus (T1DM) is a chronic autoimmune condition characterized by the irreversible destruction of the β cells of the pancreas, which leads to a lifelong dependency on exogenous insulin. Despite the advancements in insulin delivery methods, the suboptimal outcomes of these methods have triggered the search for therapies that may prevent or reverse the disease. Given the autoimmune aetiology of T1DM, therapies counteracting the immune-mediated destruction of the β-cells are the obvious target. Although several treatment strategies have been attempted to target cellular, humoral and innate immunity, very few have had a clinically meaningful impact. Of all the available immunomodulatory agents, cluster of differentiation (CD) 3 antibodies have exhibited the most promising preclinical and clinical results. Muromonab-CD3, which also happened to be a murine CD3 antibody, was the first monoclonal antibody approved for clinical use and was primarily indicated for graft rejection. The adverse effects associated with muromonab-CD3 led to its withdrawal. Teplizumab, a newer CD3 antibody, has a better side-effect profile because of its humanized nature and non-Fc-receptor-binding domain. In November 2022, teplizumab became the first immunomodulatory agent to be licensed by the US Food and Drug Administration for delaying the onset of T1DM in high-risk adults and children over 8 years old. The mechanism seems to be enhancing regulatory T-cell activity and promoting immune tolerance. This article reviews the mechanism of action and the clinical trials of teplizumab in individuals with T1DM or at risk of developing the disease.

Type 1 diabetes mellitus (T1DM) is an autoimmune disease secondary to the destruction of the insulin-producing β cells of the islets of the pancreas. Environmental factors presumably trigger the disease in genetically susceptible individuals, leading to a lifetime dependency on exogenous insulin.^1^ The risk of T1DM in the general population is 0.4% but increases to 1–9% in offspring and 6–7% in siblings.^2–7^ The concordance rate in identical twins is estimated to be 30–70%.^8–10^ Almost 50% of the familial aggregation has been attributed to the human leukocyte antigen (HLA) region on chromosome 6p21, in which the presence of HLA DR3-DQ2 and HLA DR4-DQ8 haplotypes poses a strong risk of developing T1DM.^11^ The appearance of autoantibodies against islet antigens, such as glutamic acid decarboxylase-65 (GAD-65), islet antigen 2 (IA2), zinc transporter 8 (ZnT8), islet cell and insulin, predates the development of T1DM. Autoantibodies act as robust markers of T1DM predisposition and help to categorize cohorts who might benefit from intervention.^12–14^

The islet autoantibodies suggest a role for B cells in the pathogenesis of T1DM; however, β-cell destruction is ultimately engendered by the activation of cytotoxic CD8+ T cells.^15,16^ An early step in the pathogenesis of T1DM involves the presentation of antigens, including insulin B-chain peptide (11-23) and other components of β-cell secretory granules bound to major histocompatibility complex (MHC) class I and II on antigen-presenting cells (APCs) to the cluster of differentiation (CD) 4+ T-helper cells.^17^ The activated CD4+ T-helper cells stimulate the effector cytotoxic CD8+ T cell to destroy the islet β-cells. Regulatory T cells (Tregs) counteract the autoreactive T cells to maintain peripheral tolerance and immune homeostasis; an imbalance between the two leads to a breakdown of peripheral tolerance and, eventually, to the development of T1DM.^18^

By the time T1DM is diagnosed, the pathophysiological process has progressed to damage most of the β cells of the pancreas. The invariable outcome of this process is a dependency on exogenous insulin. Despite the tremendous advancements in insulin delivery modes that have occurred over the last few decades, life expectancy is still lower in individuals with T1DM than in the general population.^19^ As a result, clinical trials targeting innate and adaptative immunity (both T and B cells) to modify the natural course of T1DM have continued to garner attention. However, most trials targeting recent-onset T1DM have had suboptimal outcomes, presumably because the β cells are already at an advanced stage of destruction and not amenable to immunomodulation (*Table 1*).^20–31^ Although the results of most studies involving participants at preclinical stages of T1DM have also not been found to be clinically relevant, some promise has been shown by teplizumab, an anti-CD3 monoclonal antibody.^31^

**Table 1:** Major clinical trials for the prevention and treatment of type 1 diabetes mellitus

Author (year)	Drug/regimen	Type of drug	Trial design	Results
**Immunomodulatory agents**
	Cook et al. (1989)^20^	Azathioprine	Anti-inflammatory and inhibitor of purine/DNA synthesis	Double-blinded controlled trial in patients with newly detected T1DM aged 2–20 years	No difference in fasting C-peptide response at 12 months between placebo and azathioprine group
	Gottlieb et al. (2010)^21^	Mycophenolate mofetil and daclizumab	Anti-proliferative agent (mycophenolate mofetil) and IL-2 receptor monoclonal antibody (daclizumab)	Multicentre, randomized, placebo-controlled, doublemasked trial for subjects diagnosed with T1DM within the previous 3 months	Mean C-peptide AUC at 2 years was unaffected by mycophenolate mofetil alone or with daclizumab versus placebo
	Bougnères et al. (1990)^22^ Christie et al. (2002)^23^	Cyclosporin	Inhibitor of T-cell activation and calcineurin with anti-inflammatory action^23^	Placebo-controlled, doubleblinded trial in children aged 7–15 years	Limited duration of remission: decreased insulin dosage, better glycaemic control and C-peptide levels two-to threefold higher than the control group
	**Agents targeting innate immunity**
Mastrandrea et al. (2009)^24^		Etanercept	TNF-α inhibitor and anti-inflammatory agent	Pilot randomized, placebo-controlled, double-blind study in subjects aged 3–18 years with T1DM, GAD-65 and/or islet cell antibody positivity, HbA1c >6% at diagnosis	The percent change in C-peptide AUC from baseline to week 24 showed a 39% increase in the etanercept group and a 20% decrease in the placebo group (p<0.05)
Moran et al. (2013)^25^		Anakinra and canakinumab	Human IL-1 receptor antagonist (anakinra) and human monoclonal anti-I L-1 antibody (canakinumab)	Randomized, placebo-controlled, double-blinded multicentre study	Statistically nonsignificant C-peptide AUC between the canakinumab and the placebo group (p=0.86) and between the anakinra and the placebo group (p=0.71)
**Agents targeting the adaptive immune system: B-Cell**	
		Pescovitz et al. (2009)^26^	Rituximab	B-l ymphocyte-depleting agent	Randomized, placebo-controlled, double-blind trial of patients with newly diagnosed T1DM aged 8–40 years receiving 4 doses of rituximab infusion on days 1, 8, 15 and 22	Mean AUC for C-peptide was higher in rituximab group at the end of 1 year. More adverse events were attested in the rituximab group
	**Self-antigen-based therapy**
		Wherret et al. (2011)^27^	GAD-65 immunotherapy	Antigen-based therapy	Multicentre, randomized, doubleblind, placebo-controlled trial of patients with recently diagnosed (<100 days) T1DM aged 3–45 years receiving 3 treatments (3 injections of 20 μg GAD-alum, 2 injections of 20 μg GAD-alum and 1 of alum, or 3 injections of alum)	Statistically nonsignificant C-peptide AUC difference between the groups
		Raz et al. (2001)^28^	DiaPep277	60 kDa hsp60 self-antigen-based therapy	Randomized, double-blind, phase II study of peptide treatment in patients with newly diagnosed (<6 months) T1DM	At 10 months, mean C-peptide concentrations fell in the placebo group, but were maintained in the DiaPep277 group. The need for exogenous insulin was higher in the placebo group
**Targeting adaptive immunity: T-cell**	
		Orban et al. (2011)^29^	Abatacept (CTLA-4 Ig)	Costimulation modulator	Randomized, double-blind, placebo-controlled trial of patients aged 6–45 years recently diagnosed with T1DM	Mean AUC for C-peptide in abatacept group was significantly higher than placebo after 2 years
	Keymeulen et al.(2021)^30^	Otelixizumab	Anti-CD3 monoclonal antibody	Multicentre, randomized, placebo-controlled trial of patients with newly diagnosed (<32 days) T1DM aged 16–27 receiving placebo or otelixizumab in one of four dose cohorts (9, 18, 27 or 36 mg over 6 days)	Residual β-cell function was better maintained with otelixizumab than with placebo at 6, 12, and 18 months, though transient symptoms of Epstein–Barr viral mononucleosis were more common
	TrialNet Study by Herold et al. (2019)^31^ (other teplizumab trials discussed in Table 2)	Teplizumab	Anti-CD3 monoclonal antibody	Randomized, placebo-controlled, double-blind phase II trial of relatives of patients with T1DM at risk of developing the disease receiving a single 14-day course of teplizumab or placebo	Median time to diagnosis of T1DM was 48.4 months in the teplizumab group and 24.4 months in the placebo group

*AUC = area under the curve; CD3 = cluster of differentiation 3; CTLA-4 Ig = cytotoxic T lymphocyte-associated antigen-4-Ig; GAD = glutamic acid decarboxylase; HbA1c = glycated haemoglobin; hsp60 = heat shock protein 60; IL = interleukin; kDa = kilodalton; T1DM = type 1 diabetes mellitus; TNF = tumour necrosis factor.*

Teplizumab is the only drug to date to be approved by the US Food and Drug Administration (FDA) to delay the onset of T1DM in relatives of individuals at risk of developing the disease and finds a place in clinical application.^32–34^ Teplizumab use could lead to a transient systemic rise in the percentage of CD4+Foxp3+ T cells, thereby restoring the imbalance between tolerogenic Tregs and pathogenic T-cells.^35^ Teplizumab is also being studied to treat recent-onset T1DM.^36,37^ This article reviews teplizumab's mechanism of action and clinical trial data and its potential use in various stages of T1DM.

## Literature search strategy

For our literature review, we searched PubMed, Scopus, Cochrane Library and Google Scholar for articles published in English before 30 May 2023. The search terms were a combination of “teplizumab”, “anti-CD3 therapy”, “CD3 antibody” and “immunotherapy” with “type 1 diabetes”. The abstracts were evaluated for relevance, and the full text of all relevant articles was retrieved. The references of the selected papers were also scrutinized.

## Pathophysiology of type 1 diabetes mellitus

### Genetics of type 1 diabetes mellitus

T1DM is an autoimmune disorder secondary to the destruction of β-cells in the pancreatic islets of Langerhans. Genome-wide association studies revealed that the polymorphisms of genes responsible for antigen presentation and immune response, including those in HLA 6p21 and non-HLA 16p11.2 *loci*, are linked to the pathogenesis of T1DM.^38^

### Environmental triggers

Various environmental triggers have been implicated in the pathogenesis of T1DM. Environmental triggers include perinatal factors such as higher maternal age, increased birth weight, preeclampsia, neonatal respiratory distress, neonatal jaundice secondary to ABO incompatibility; and viral infection-induced autoimmunity or molecular mimicry, childhood vaccination and early exposure to cow's milk or cereals.^39–43^ Toxins found in water and food, such as nitrates and nitrosamines, are also associated with the induction of autoimmunity and the development of T1DM.^41,44,45^ Adequate vitamin D supplementation and omega-3 fatty acids are considered to be protective against T1DM.^46,47^

### Autoantigens, B cells and inflammatory cytokines

The release of β-cell autoantigens can be caused by exogenous triggers or by an endogenous β-cell defect. The released antigens are then engulfed and presented on the surface of APCs with MHC class II. In the presence of costimulatory molecules (CD80/86 on T cells and CD28 on APCs), autoreactive CD4+ T cells are activated, inducing the release of cytokines such as interferon-γ and interleukin (IL)-2 for the recruitment of cytotoxic CD8+ T cells.^48,49^ Interferon-γ, tumour necrosis factor (TNF) α and IL-1 are the major cytokines produced by these pathogenic T-cells that lead to insulitis and β-cell destruction.^50,51^ B-cell activation leads to the formation of antibodies against various islet autoantigens, such as GAD-65, IA2, ZnT8, islet cells and insulin, which act as biomarkers for T1DM.^12,15^

### The role of cellular immunity

**Figure 1:**
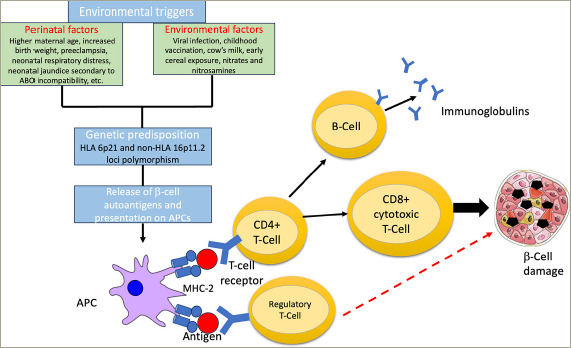
Pathophysiology of the development of type 1 diabetes mellitus

Cellular immunity plays a prominent role in the pathogenesis of T1DM. During the development of the immune system, potentially dangerous T-l ymphocytes in the thymus and B-l ymphocytes in the bone marrow undergo negative selection. Cells that escape this mechanism of central tolerance are subjected to peripheral tolerance, where they are either neutralized or suppressed. Tregs play a crucial role in the immune response network for peripheral tolerance.^52^ CD4+4+CD25+Foxp3+ cells originating in the thymus are natural Tregs, whereas induced Tregs are derived from active T cells in the periphery.^53^ Induced Tregs suppress the effector T cells through the inhibitory cytokines transforming growth factor-β and IL-10 and maintain immune homeostasis to achieve a balance between the pro-and the anti-inflammatory responses.^35^ *Figure 1* is a simplified depiction of the pathophysiology of T1DM.

### The role of regulatory T cells

A quantitative or a qualitative defect in Tregs can lead to the development of T1DM.^54^ For example, the balance between effector T cells and Tregs may be altered by genetic polymorphisms in the IL-2 receptor pathway. The ensuing defect in the IL-2/IL-2 receptor signalling pathway can hamper the T-effector and Treg crosstalk, and lead to the proliferation of autoreactive effector T cells that disrupt immune homeostasis.^55^ The Tregs in T1DM secrete a disproportionate amount of the proinflammatory cytokines IL-12 and IL-1 8.^56^ In *ex vivo* and *in vitro* studies of Tregs in individuals with T1DM, lower frequency of glucocorticoid-induced TNF receptors family-related protein was shown to increase the susceptibility to apoptosis.^57^ Additional evidence has shown that CD4+Foxp3+ Tregs, a specialized subset of helper T cells, are dysfunctional in T1DM.^58^

### Enhancing regulatory T-cell activity as a therapeutic target

Boosting Treg proliferation and activity has emerged as a promising target for blocking the T1DM pathogenic pathway. Of the several agents that have been tried, the anti-CD3 monoclonal antibody seems to be the most favourable for boosting Treg proliferation and activity.^59^ Anti-CD3 antibodies preserve Tregs and diminish pathogenic T cells, restoring immune tolerance. The administration of anti-CD3 antibodies results in a transient systemic rise in the percentage of CD4+Foxp3+ T cells and in a reduction of CD4+Foxp3-conventional T cells.^60^ Although the mechanism of action of anti-CD3 therapy in regulating T1DM pathogenesis is only partially understood, it seems that the predominant effect of anti-CD3 therapy is to enhance Treg activity.

## Stages and progression of type 1 diabetes mellitus

The three stages in the evolution of T1DM are the following: stage 1 is characterized by β-cell autoimmunity, normoglycaemia and absence of symptoms; stage 2 refers to progression to dysglycaemia but is still presymptomatic; and stage 3 is marked by the appearance of symptoms.^61^ Environmental factors trigger cellular and humoral immune responses in individuals who are genetically predisposed, initiating stage 1, marked by the presence of two or more islet autoantibodies.^62^ The risks of the disease transitioning to stage 2 and progressing to overt stage 3 increases with the number of detectable antibodies.^14,63^ Defining these stages helps to identify at-risk individuals and categorize participants for T1DM prevention trials.^61^

## Targets for immune intervention in type 1 diabetes mellitus

Several small interventional trials were conducted in the last two decades to assess the effect of nonspecific immunosuppressants, such as azathioprine and cyclosporine, on the autoimmune pathways of T1DM pathogenesis.^64,65^ Although these therapies showed promise, their effect waned once the drugs were tapered. With the discovery of autoantigens in T1DM, it was hypothesized that administering these antigens could create a tolerogenic response. Agents such as oral and nasal insulin, heat shock protein 60 (Hsp60) and GAD-65 were tried but had minimal success. 27,28,66–68

Humoral immunity has also been tested as a preventive strategy for T1DM. A phase II clinical trial demonstrated modest success with rituximab, an anti-CD20 monoclonal antibody that interferes with the B-cell-mediated activation of T cells (ClinicalTrials.gov identifier: NCT00279305).^26^ Preclinical studies suggested that neutralizing proinflammatory and Th1-cytokines such as TNF-α and IL-1β (the mediators of inflammatory damage to the β cells) could modulate the pathogenesis of T1DM.^69,70^ As a result, drugs such as etanercept (a TNF-α blocker), anakinra (an IL-1 inhibitor) and canakinumab (a human monoclonal antibody targeting IL-1β) were investigated but did not have a clinically meaningful response.^24,25,71^

Animal studies showed that Tregs play a dominant role in promoting immune tolerance in T1DM.^72–74^ Various drug classes have been tried to inactivate, deplete or modulate T-cell function. Further activation of T cells requires costimulatory peptides after antigen-binding to MHC molecules on APCs. Abatacept (CTLA-4-I g) induces costimulation blockade, thereby preventing autoreactive T-cell activation. In a phase II clinical trial (ClinicalTrials.gov identifier: NCT00505375), abatacept slowed the decline of β-cell function when injected in individuals with newly diagnosed T1DM.^29^ The depletion of the T cells by rabbit antisera antithymocyte globulin was found to reverse T1DM only in preclinical studies.^75–77^ Daclizumab, a humanized monoclonal antibody that targets CD25+ T cells in combination with mycophenolate mofetil, was not effective in arresting β-cell destruction in individuals with newly diagnosed T1DM.^21^ Haematopoietic stem cell transplantation was investigated to provide a period free from T-cell influence for the maturation of new lymphocyte progenitor cells without the recruitment of anti-self-activity; however, this procedure has unclear benefits.^78^ T-cell modulation by an LFA3-I g protein, alefacept, exhibited a favourable Treg/T-effector ratio by selective depletion of autoreactive T cells.^79^ However, the most promising immunomodulatory therapy currently is the anti-C D3 monoclonal antibody teplizumab. The predominant mechanism of teplizumab seems to be the upregulation of Tregs and the promotion of immune tolerance. Major immune intervention-based clinical trials in T1DM are summarized in *Table 1*.^20–31^

## Anti-CD3 monoclonal antibodies

Muromonab-CD3 (Orthoclone OKT3®; Janssen-Cilag, Raritan, NJ, USA), a murine immunoglobulin G2a antibody, was the first monoclonal antibody to be approved for human use to prevent renal allograft rejection.^80^ Although effective, its clinical use was shown to be limited, as most recipients experienced a flu-l ike syndrome, with fever, chills, headache and pulmonary oedema developing within hours of treatment.^81^ Muromonab-CD3 caused reversible loss of CD3 T-cell receptor (TCR) expression and rendered the cell immunoincompetent.^82^ The drug's adverse effects resulted from the release of inflammatory cytokines by T cells triggered by the cross-l inking of TCR and CD3. Further, binding by Fc-receptor (FcR) bearing cells, such as monocytes, to the Fc portion of muromonab-CD3 was found to increase this cross-l inking and worsened the severity of cytokine release syndrome.^83^

FcR-nonbinding CD3 antibodies that induce less cytokine release have been developed. Additionally, humanized antibodies are preferred, as murine antibodies tend to lose efficacy due to the development of antibodies to the murine component.^84^ Visilizumab (PDL BioPharma, Inc., Reno, NV, USA), otelixizumab (GSK, Brentford, UK), teplizumab (Provention Bio, Red Bank, NJ, USA), foralumab (Tiziana Life Sciences, London, UK) are the most recent humanized non-FcR binding antibodies; they are better tolerated and have shown promise in a variety of inflammatory and autoimmune conditions.^84,85^

**Figure 2:**
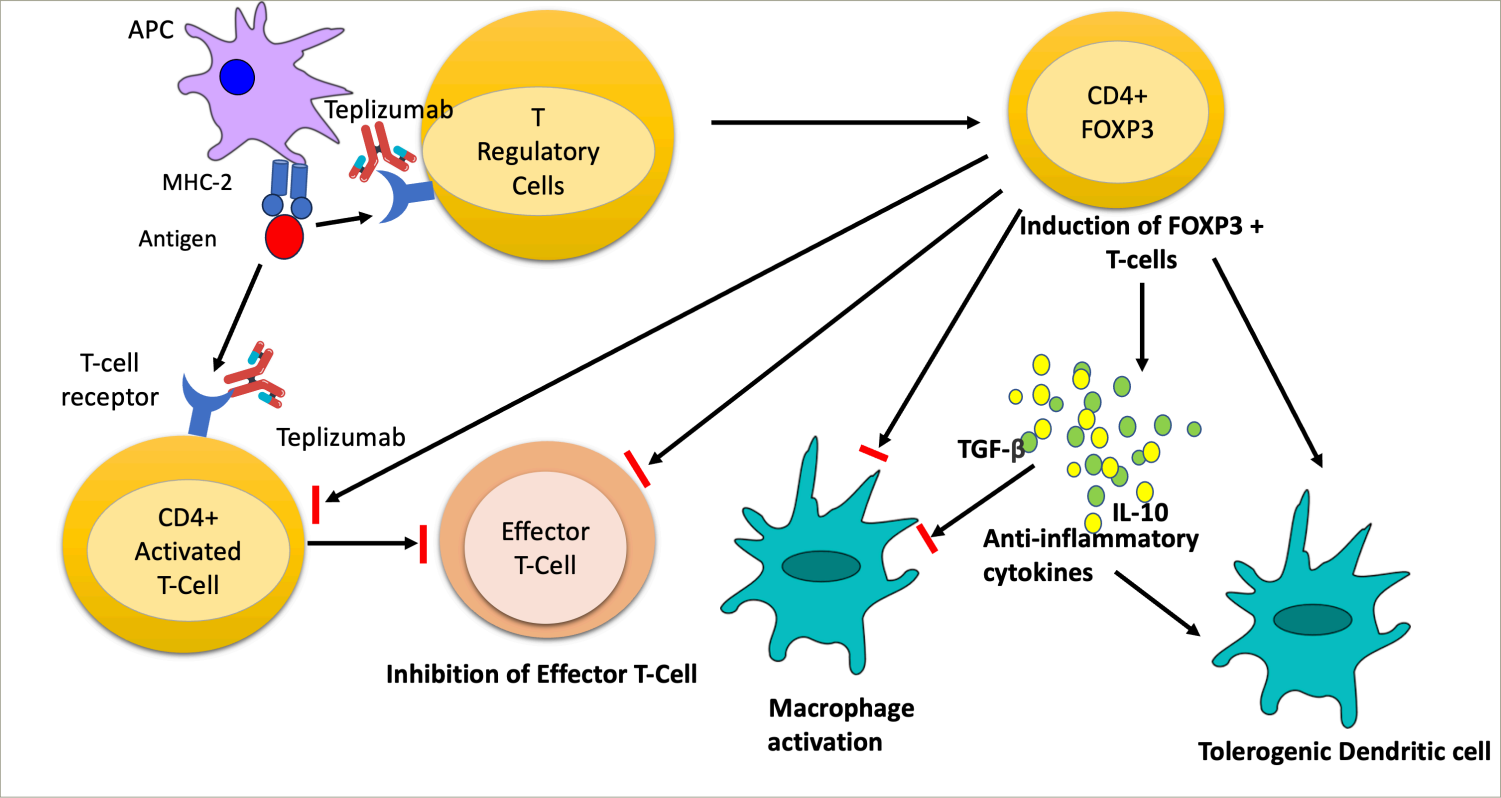
Proposed mechanism of action of teplizumab

Teplizumab, or hOKT3 γ1(Ala-Ala), a humanized version of the muromonab-CD3 antibody, retains the same binding region of muromonab-CD3 but has undergone alanine substitutions that reduce Fc binding in crucial positions.^86^ The proposed mechanism of action of teplizumab is depicted in *Figure 2*. Otelixizumab, or ChAglyCD3, is another humanized version of the muromonab-CD3 antibody; however, it lacks the glycosylation site in the Fc portion, which results in decreased Fc binding.^87^ Otelixizumab binds to CD3ɛ molecules, a protein-forming part of the CD3ɛ/ TCR on the surface of T lymphocytes, and causes their downmodulation. This translates into enhanced Treg response and could potentially prevent β-cell destruction.^88^ The dose-l imiting adverse effect of otelixizumab is Epstein-Barr virus reactivation.^30^ Of the two, teplizumab has been studied in more clinical trials and has a better safety profile than otelixizumab.^85^

## The history of the development of teplizumab

Teplizumab was first developed at the University of Chicago in partnership with Ortho Pharmaceuticals (Cypress, TX, USA).^89^ It was then further developed by MacroGenics Inc. (Rockville, MD, USA) in collaboration with Eli Lilly and Company (Indianapolis, IN, USA), who investigated the drug's ability to reduce insulin need and improve glycated haemoglobin (HbA1c) during the phase III Protégé trial (The Protégé study -clinical trial of MGA031 in children and adults with recent-onset type 1 diabetes mellitus; ClinicalTrials.gov identifier: NCT00385697).^37^ After the drug failed to meet this primary endpoint, further clinical trials were abandoned by this group. The rights to the drug were later acquired by Provention Bio, who carried out subset analyses within the original study and identified its potential to delay the progression of T1DM. This hypothesis was confirmed in subsequent randomized controlled trials (RCTs).^31^ Teplizumab was granted a breakthrough therapy designation by the FDA and was the first drug to receive approval for the prevention of T1DM in at-risk individuals.^33,34,90^

## Preclinical and animal model studies

The nonobese diabetic (NOD) mouse strain represents a reliable animal model of T1DM in humans.^91^ An early study in NOD mice suggested that insulitis could be delayed by the neonatal injection of a CD3 antibody.^92^

A low dose of the hamster anti-CD3 monoclonal antibody 145 2C11 restored self-tolerance and induced durable remission in NOD mice with overt diabetes.^93^ Data from the NOD mice model demonstrated that resetting Tregs with CD3 antibodies promotes the resolution of insulitis and the promotion of self-tolerance.^72^ These findings paved the way for exploring the role of humanized non-FcR binding CD3 antibodies in human clinical trials.

## Teplizumab trials in established type 1 diabetes mellitus

### Phase II trials

Several human clinical trials have shown that teplizumab is an effective strategy for postponing the drop in C-peptide production and helping to maintain β-cell function.^31,36,94^

In a pioneering study by Herold et al. in 2019, 24 participants newly diagnosed with T1DM were randomly assigned to receive either teplizumab or placebo.^31^ Treatment within 6 weeks of diagnosis with a single course of antibodies led to maintained or improved insulin production after 1 year (p=0.01). The treatment effect on insulin response lasted 12 months after diagnosis. Mild-to-moderate side effects were observed, including arthralgia, fever, nausea, headache, vomiting and rash. Long-term adverse effects were not observed.^94^

Another open-l abel RCT showed a similar positive impact of teplizumab in new-onset T1DM.^36^ Teplizumab was found to preserve insulin production and reduce exogenous insulin use in some patients aged between 8 and 30 years with new-onset T1DM for up to 2 years after entering the study. Teplizumab use resulted in a reduced C-peptide decline at 2 years compared with controls (p=0.002). The findings indicate that the drug might preserve insulin synthesis and decrease the need for exogenous insulin in new-onset T1DM.^36^

### Phase III trials

The Protégé trial was a randomized, double-blind, parallel, placebo-controlled, phase III study that included individuals between 8 and 35 years who had been diagnosed with T1DM for 12 weeks or less. The participants were randomized 2:1:1:1 to receive one of the three regimens of teplizumab infusions (a 14-day full dose of ~9,034 μg/m^2^, a 14-day low dose of ~2,985 μg/m^2^ or a 6-day full dose of ~2,426 μg/m^2^) or placebo at baseline and then repeated at 26 weeks. The 14-day full dose of teplizumab reduced the loss of C-peptide mean area under the curve at 2 years versus placebo. The need for exogenous insulin was also reduced with teplizumab compared with placebo. Antidrug antibodies developed in 77% (318/415) of participants receiving teplizumab and in 13% (13/98) of those receiving placebo when samples were obtained at 28 or 56 days, without apparent change in efficacy. No new safety or tolerability issues were observed over the 2 years of the trial. Even though teplizumab has shown promising results in preventing disease progression, metabolic benefits, such as a significant reduction in HbA1c, have not been demonstrated.^37,95^

## Teplizumab in the prevention of type 1 diabetes mellitus

Teplizumab delays the onset of T1DM in high-risk relatives of individuals with T1DM. In a landmark phase II RCT (Teplizumab for prevention of type 1 diabetes in relatives “at-risk”; ClinicalTrials.gov identifier: NCT01030861), Herold et al. randomized 76 participants to receive either teplizumab (44) or placebo (32).^31^ Teplizumab was found to delay the progression to clinical T1DM compared with placebo. The study participants were relatives of persons with T1DM who did not have diabetes and were at high risk of developing T1DM. They had at least two positive antibodies 6 months before participating in the study, with evidence of dysglycaemia on the oral glucose tolerance test. Following a single course of a 14-day infusion of teplizumab, the median time to T1DM diagnosis was 48.4 months in the teplizumab group and 24.4 months in the placebo group. T1DM was diagnosed in 19 (43%) of the participants who received teplizumab and in 23 (72%) of those who received placebo (hazard ratio for the diagnosis of T1DM [teplizumab versus placebo] 0.41, 95% confidence interval 0.22–0.78; p=0.006). The annualized rate of diagnosis of T1DM significantly differed in the teplizumab group (14.9% per year) versus placebo (35.9% per year). The rate of infection was found to be similar between the two groups. There were expected adverse events of rash (36% in the teplizumab group) and transient lymphopenia.^31^

*The induction of T-regulatory cells by teplizumab prevents the activation of CD4+T-cells and cytotoxic CD8+ cells. The induction of CD4+Foxp3+ T-cells leads to the secretion of anti-inflammatory cytokines tissue growth factor-p and interleukin-10 and restore the balance between anti-inflammatory and pro-inflammatory stimuli.*

*APC = antigen-presenting cell; CD4 = cluster of differentiation 4; F0XP3 = forkhead box protein 3; HLA = human leukocyte antigen; IL-10 = interleukin-10; MHC = major histocompatibility complex; TGF-p = tissue growth factor-p,*

**Table 2:** Summary of important clinical trials of teplizumab

Author or study (year)	Type of study	Results
Herold et al. (2002)^94^	Phase I/II trial RCT	Treatment within 6 weeks of diagnosis of T1DM with a single course of monoclonal antibodies led to maintained or improved insulin production after 1 year
The AbATE study (2013)^36^	Open-l abel, phase II RCT	Teplizumab preserved insulin production and reduced the requirement for exogenous insulin in patients with new-onset T1DM
The Protégé study (2013)^37,95^	Double-blind, parallel, placebo-controlled, phase III RCT	Teplizumab therapy reduced C-peptide loss and need for exogenous insulin untill 2 years after diagnosis
Sims et al. (2021)^96^	RCT of relatives of subjects with T1DM who are not diabetic and are at high risk of developing T1DM	Treatment with teplizumab enhanced β-cell activity in the participants

*RCT = randomized controlled trial; T1DM = type one diabetes mellitus.*

In an extended follow-up analysis of this trial (median 923 days), Sims et al. reported that the efficacy of teplizumab's initial 2-week treatment course persisted.^96^ The median times to diagnosis were 59.6 and 27.1 months for the teplizumab and the placebo group, respectively. Half of the participants treated with teplizumab were diabetes free compared with 22% of participants in the placebo group (hazard ratio 0.457; p=0.01). Furthermore, teplizumab improved insulin production, which was measured using the C-peptide response, and β-cell function. C-peptide response was associated with increased exhaustion of effector CD8+ T-l ymphocytes, which also secreted fewer proinflammatory cytokines.^96^ Therefore, it was concluded that teplizumab delays the progression to clinical T1DM in high-risk individuals.

These positive findings were further corroborated by data from a metanalysis conducted by Nourelden et al. to assess the safety and efficacy of teplizumab for the treatment of T1DM.^97^ Eight randomized clinical trials involving 866 patients found that subjects treated with teplizumab had lower insulin use and higher area under the curve of C-peptide compared with placebo. However, teplizumab was not found to significantly alter HbA1c levels at any time. Teplizumab was associated with side effects such as skin and subcutaneous tissue disorders and lymphopenia.^97^ The major clinical trials of teplizumab are described in *Table 2*.^36,37,94–96^

## On-going trials of teplizumab

### The phase III trial PROTECT

Provention Bio is currently evaluating teplizumab in individuals with newly-diagnosed T1DM in the global, multicentre, phase III study PROTECT (Recent-onset type 1 diabetes trial evaluating efficacy and safety of teplizumab; ClinicalTrials.gov identifier: NCT03875729).^98^ The trial randomized 2:1 approximately 300 children and adolescents with recent-onset T1DM between the ages of 8 and 17 to receive two 12-day courses of either teplizumab or placebo 6 months apart. The drug was administered within 6 weeks of diagnosis. The primary efficacy endpoint is change in C-peptide from baseline. Secondary endpoints are insulin use, HbA1c, episodes of hypoglycaemia and adverse events. The company expects topline data in the second half of 2023.^98,99^

### The TrialNet 10 extension study

The on-going TrialNet (TN) 10 extension study (At-risk for type 1 diabetes extension study; ClinicalTrials.gov identifier: NCT04270942) was designed as an extension of the original AT-Risk type 1 diabetes study sponsored by the National Institutes of Health (Teplizumab for prevention of type 1 diabetes in relatives “at-risk”; ClinicalTrials.gov identifier: NCT01030861).^100,101^ It is a single-arm, multicentre, open-l abel trial involving the administration of a 12-day course of intravenous teplizumab to participants treated with teplizumab and those treated with placebo from the original study who went on to develop T1DM, with a target of 30 participants. The study aims to determine whether administering teplizumab within 1 year of diagnosis of T1DM reduces β-cell loss. The participants of this study will also be followed up for 78 weeks to assess the safety and tolerability of the drug. The expected trial completion date is August 2024.^101^

## Clinical implications and limitations of teplizumab in current diabetes care

On 17 November 2022, teplizumab became the first immunoprevention agent to be approved by the FDA for delaying the onset of T1DM in adults and children aged 8 years and older.^34^ Delaying T1DM onset offers benefits for patients, such as reduced insulin usage and hospital visits, fewer complications, better glucose control, and extended time for potential interventions.^102^

Despite this approval, teplizumab faces two main challenges. First, teplizumab's licensing is limited to a mostly unidentified group, as there are no widespread screening programs for early-stage T1DM in the general population except for research scenarios. To overcome this limitation, we require a screening program to identify at-risk individuals and educate healthcare providers about T1DM stages and the at-risk population. The evidence for general population autoantibody screening is questionable, and optimal screening methods are not defined.^103^ Even for the few potential candidates, the integration of screening, monitoring and therapy into clinical T1DM care needs to be investigated. Second, the high treatment cost, approximately US$200,000 per course, is also a great hindrance.^104^ Despite these challenges, teplizumab opens the door for exploring immunomodulation as a potential therapy for T1DM.

Safety evidence about the drug's impact on children and adolescents is an important consideration. Precautions such as premedication, monitoring for cytokine release syndrome, temporary lymphopenia risk, severe infection risk and hypersensitivity reactions are included in the licence. Age-appropriate vaccinations are necessary before starting treatment. It is important to note that teplizumab does not cause longterm immunosuppression, as T-cell levels return to normal in 6–8 weeks. Safety data up to 7 years show infection rates did not increase beyond the initial infusion phase. No data are available to suggest higher cancer risk.^105^ A clinical trial evaluating teplizumab for treating established cases of T1DM is currently on-going (Type 1 diabetes extension study [T1DES]; ClinicalTrials.gov identifier: NCT02734277).^106^

## Future directions and research

Despite the enormous progress in our understanding of the pathophysiology of T1DM, several unanswered questions remain. Although teplizumab delays the start of T1DM by nearly 3 years, it is unclear whether a second course of the medication at this point would further delay onset.

Extending the eligible age range for therapy is another area of investigation – teplizumab's FDA clearance was based on the PREVENTION study, in which the youngest participant was 8 years old. However, it is well known that children as young as 3 years or less can develop T1DM and may benefit the most from delayed disease onset.^31^ T1DM is a heterogeneous group of disorders, and its phenotype may vary with the age of presentation. Individuals who present earlier in childhood tend to have more severe disease, progress more rapidly and lose their ability to produce endogenous insulin earlier.^107^ Studies have shown that these differences in presentation, age of onset and clinical course may be due to variations in the primary autoantibody involved. The understanding of patterns of antibody involvement and its therapeutic and prognostic significance will further help to triage individuals at higher risk of T1DM.

Combination treatment might broaden the potential for T1DM prevention. Teplizumab may be used in synergy with other medications that work through different mechanisms to delay T1DM. One example of such medications is verapamil (Pfizer, New York, NY, USA), which has been recently reported to preserve β-cell function in new-onset T1DM.^108,109^ Going forward, it may also be crucial to explore RCTs studying potential responders as opposed to nonresponders and analyzing the involved biomarkers.

## Conclusions

The recent FDA approval of teplizumab for delaying the onset of T1DM in high-risk individuals heralds an exciting new era in diabetes research and clinical care. The clinical use of teplizumab is currently limited to relatives of individuals of T1DM with dysglycaemia who have two or more islet autoantibodies but have not yet reached the stage of overt diabetes. The administration of this therapy in individuals with recently diagnosed T1DM is shown to preserve β-cells but does not translate to an improvement in HbA1c. Even then, the formal approval of teplizumab for the prevention of T1DM and an understanding of the critical role of Tregs in promoting immune tolerance are critical breakthroughs that will stimulate further research.
